# Health-related quality of life and symptoms in patients with rituximab-refractory indolent non-Hodgkin lymphoma treated in the phase III GADOLIN study with obinutuzumab plus bendamustine versus bendamustine alone

**DOI:** 10.1007/s00277-016-2878-5

**Published:** 2016-11-30

**Authors:** Bruce D. Cheson, Peter C. Trask, John G. Gribben, Natalie Dimier, Eva Kimby, Pieternella J. Lugtenburg, Catherine Thieblemont, Elisabeth Wassner-Fritsch, Aino Launonen, Laurie H. Sehn

**Affiliations:** 1 Lombardi Comprehensive Cancer Center, Georgetown University Hospital, 3800 Reservoir Road, NW, Washington, DC 20007 USA; 2 Genentech, Inc., 1 DNA Way, South San Francisco, CA 94080 USA; 3 Barts Cancer Institute, Queen Mary University of London, Charterhouse Square, London, EC1M 6BQ UK; 4 Roche Products Ltd, Hexagon Place, 6 Falcon Way, Shire Park, Welwyn Garden City, AL7 1TW UK; 5 Karolinska University Hospital, SE-14186 Stockholm, Sweden; 6 Erasmus MC Cancer Institute, Wytemaweg 80, 3015 CN Rotterdam, The Netherlands; 7 AP-HP Hôpital Saint-Louis, 1 Avenue Claude Vellefaux, 75010 Paris, France; 8 F. Hoffmann-La Roche Ltd, Grenzacherstrasse 124, CH-4070 Basel, Switzerland; 9 British Columbia Cancer Agency and the University of British Columbia, 675 West 10th Avenue, Vancouver, BC V5Z 1L3 Canada

**Keywords:** Follicular lymphoma, FACT-Lym, GADOLIN, Non-Hodgkin lymphoma, Obinutuzumab, Quality of life

## Abstract

We present health-related quality of life (HRQoL) data from GADOLIN, comparing bendamustine (B) alone or combined with obinutuzumab (G-B) in rituximab-refractory indolent non-Hodgkin lymphoma patients. The Functional Assessment of Cancer Treatment-Lymphoma (FACT-Lym) questionnaire was administered on day 1 of cycles 1, 3, and 5 during treatment, at end of induction (EOI), bi-monthly for 2 years during maintenance/follow-up, and annually during extended follow-up until progression/death. Time to first ≥6-point worsening from baseline in the FACT-Lym trial outcome index (TOI) was estimated. Minimally important differences at individual subscale and total score level were used to define the proportion of patients reporting improvement on the FACT-Lym lymphoma-specific subscale (≥3 points), FACT-Lym TOI (≥6 points), and FACT-Lym total score (≥7 points). Overall, 396 patients were randomized. Analysis was conducted when 175 Independent Review Committee-assessed progression-free survival (PFS) events were observed. Questionnaire completion rates were generally balanced between arms at baseline, EOI, and final follow-up. Median time to ≥6-point worsening from baseline on the FACT-Lym TOI was 8.0 months in the G-B arm and 4.6 months in the B arm (HR 0.74; 95% CI 0.56–0.98). More G-B patients reported meaningful improvements on the FACT-Lym questionnaire subscales. Results were similar when follicular lymphoma patients were analyzed separately. The delayed time to worsening and greater proportion of patients reporting meaningful improvement in HRQoL in the G-B arm suggest that benefit in PFS is not at the expense of an increase in treatment-related toxicity that could lead to reduced HRQoL.

**Electronic supplementary material:**

The online version of this article (doi:10.1007/s00277-016-2878-5) contains supplementary material, which is available to authorized users.

## Introduction

Indolent non-Hodgkin lymphoma (iNHL)—including follicular lymphoma (FL) and non-follicular histologies—is mostly slow growing, but rarely cured. FL accounts for approximately 70% of iNHL and 22% of all NHL [1]. Despite significant improvements in response rates and progression-free survival (PFS) with the addition of rituximab to chemotherapy [2–6], iNHL patients continue to relapse or become refractory to treatment resulting in limited treatment options.

Bendamustine (B) has been shown to be effective in rituximab-refractory (R-refr) patients (75–77% overall response rate), but median PFS is short [7, 8]. Obinutuzumab (GA101; Gazyva/Gazyvaro; G) is a novel, humanized, glyco-engineered type II anti-CD20 monoclonal antibody, developed to have superior efficacy compared with rituximab. In early clinical trials, G demonstrated encouraging efficacy in relapsed iNHL patients, and responses were observed in R-refr patients [9–11]. In the phase III GADOLIN study evaluating B with or without G in patients with R-refr CD20+ iNHL, prolonged PFS in the G-B arm was demonstrated [12].

Equally important to improving efficacy outcomes in these patients is understanding the impact of treatment on disease- and treatment-related symptoms and function. Relatively little is known about the health-related quality of life (HRQoL) of relapsed/refractory (Rel/Refr) and R-refr iNHL patients. The majority of information comes from cross-sectional studies of long-term survivors of NHL that have combined different indolent subtypes and required patients to be several months from receipt of chemotherapy [13–15]. In a proportion of these studies, partly because many iNHL patients are asymptomatic at presentation, the HRQoL of iNHL patients is akin to that of the general population. Other studies have highlighted that disease-related symptoms, such as lymphadenopathy, fatigue, and disease-related B symptoms (e.g., weight loss, fever, and night sweats), have a negative impact on patients [14, 15].

A study by Pettengell reported HRQoL in five categories of FL patients, including one category classified as “active disease-relapsed” [13]. Although it was a cross-sectional study and unrelated to specific treatment, this study identified that patients with active relapsed disease had the lowest scores on aspects of HRQoL (physical well-being, PWB; functional well-being, FWB; emotional well-being, EWB; and social/family well-being, SWB) and lymphoma-related symptoms. Information on the impact of disease and treatment-related symptoms on the HRQoL of Rel/Refr and R-refr iNHL patients, however, is limited.

In evaluating the efficacy benefit of new treatments in Rel/Refr and R-refr patients, it is important to demonstrate that any improvement in PFS occurs without adversely impacting HRQoL and that there is an increase in the time to patient-reported symptom deterioration with a new treatment. The current study evaluates the impact of G-B and B on patient-reported HRQoL, lymphoma-specific symptoms, and health status of patients with R-refr iNHL in the GADOLIN trial.

## Methods

### Trial design

GADOLIN (NCT01059630) is an open-label, phase III study of B with or without G in patients with R-refr CD20+ iNHL. Patients were randomly assigned 1:1 to the two treatment arms. B patients received 120 mg/m^2^/day given intravenously (IV) on days (D) 1 and 2 of each cycle (C), every 28 days, for up to 6 cycles. G-B patients received 90 mg/m^2^/day B given IV on D1 and 2 of C1–6. G was administered by IV infusion as an absolute dose of 1000 mg on D1, 8, and 15 of C1 and D1 of C2–6. Patients who did not progress (complete response, partial response, or stable disease, at end of induction [EOI] treatment) received G maintenance, 1000 mg IV every 2 months, until progression of disease (PD) or up to 2 years.

Eligible patients were aged ≥18 years with histologically documented, R-refr CD20+ iNHL. Patients previously treated with a maximum of four unique chemotherapy-containing treatment regimens, with an Eastern Cooperative Oncology Group (ECOG) performance status of 0, 1, or 2, were included. R-refr was defined as no response or progression within 6 months of completion of the last dose of rituximab therapy (monotherapy or combined with chemotherapy).

GADOLIN was conducted in accordance with the Declaration of Helsinki and the International Conference on Harmonization guidelines for Good Clinical Practice. All patients gave written informed consent. The protocol was approved by the ethics committees of participating centers and is registered at ClinicalTrials.gov.

### HRQoL assessments

The 42-item Functional Assessment of Cancer Treatment-Lymphoma (FACT-Lym) questionnaire was used to assess aspects of HRQoL [16, 17]. The FACT-Lym questionnaire is composed of the FACT-General (FACT-G)—a 27-item compilation of general questions scored on a 5-point scale ranging from 0 = “not at all” to 4 = “very much”—and an additional 15 items that assess patient concerns relating to lymphoma: the FACT-Lym lymphoma-specific subscale (FACT-Lym LYMS; range, 0–60). FACT-G items are divided into four primary HRQoL domains: PWB (seven items; range, 0–28), SWB (seven items; range, 0–28), EWB (six items; range, 0–24), and FWB (seven items; range, 0–28). The FACT-Lym LYMS consists of common lymphoma disease and/or treatment-related symptoms (e.g., pain, fever, swelling, night sweats, insomnia, itching, weight loss, fatigue, and loss of appetite). Three summary scales: FACT-Lym trial outcome index (FACT-Lym TOI; range, 0–116; composed of the PWB, FWB, and FACT-Lym LYMS scales); FACT-G (range, 0–108; composed of the PWB, FWB, SWB, EWB), and the FACT-Lym total score (FACT-Lym TOT, range, 0–168; composed of all of the scales) can also be calculated. Higher scores are reflective of better HRQoL.

Clinically meaningful minimally important differences (i.e., the smallest amount of change considered important to patients) at the individual subscale and FACT-Lym TOT level were pre-specified and used to define the proportion of patients reporting meaningful changes on the FACT-Lym LYMS (≥3 points), FACT-Lym TOI (≥6 points), and FACT-Lym TOT (≥7 points) as a result of treatment [18]. The FACT-Lym questionnaire was administered on D1 of C1, 3, and 5 during treatment, at EOI, bi-monthly for 2 years (where non-progressing G-B patients received G maintenance and B patients were observed), and annually during extended follow-up until PD.

### Statistical methods

FACT-Lym questionnaire analysis included all randomized patients in the intent-to-treat (ITT) population who had a non-missing baseline and at least one post-baseline patient-reported outcomes (PRO) assessment. Subgroup analyses were conducted in the FL subpopulation based on the stratified randomization for diagnosis. For missing items within the questionnaire, prorated scores were calculated according to developer guidance [19]. The percentage of participants randomized to each arm who completed the FACT-Lym questionnaire at each assessment point after baseline (PRO completion rates) was calculated and compared. For each of the FACT-Lym questionnaire scales, descriptive statistics for recorded values at each visit and changes from baseline were conducted. In addition to an evaluation of the overall treatment groups, analyses were conducted to understand if there were differences in patients who reported meaningful changes: the time it took for patients to deteriorate and the proportion of individuals who improved at each assessment point during the study. Since HRQoL was a secondary outcome of GADOLIN, and to maintain the pre-specified hierarchical testing structure intended to protect the overall type I error, hypothesis testing of HRQoL was not conducted. Results presented herein are considered exploratory in nature.

Survival function of time to earliest occurrence of ≥6-point worsening from baseline in the FACT-Lym TOI for each arm was estimated with a Kaplan-Meier curve. If patients did not have a post-baseline FACT-Lym assessment, data were censored at the time of randomization: patients who did not reach ≥6-point worsening were censored at their last completed PRO questionnaire. The proportion of patients who met the following criteria for FACT-Lym response was summarized by visit using available data: ≥3-point improvement in the FACT-Lym LYMS, ≥6-point improvement in the FACT-Lym TOI, and ≥7-point improvement in the FACT-Lym TOT.

## Results

Overall, 396 patients were randomized (194 to G-B and 202 to B [198 treated]). Baseline characteristics (e.g., proportion of males/females, ECOG performance scale, iNHL subtype, Follicular Lymphoma International Prognostic Index score, time from initial diagnosis to randomization, and presence of B symptoms) were balanced between arms. Median age was 63 years, and patients had a median of two prior therapies. Primary study analysis was undertaken when 175 Independent Review Committee (IRC)-assessed PFS events were observed. PFS was significantly longer with G-B (median not reached [NR]; 95% confidence interval [CI] 22.5 months—NR) than B (median 14.9 months; 95% CI 12.8–16.6 months); hazard ratio (HR) for progression or death was 0.55 (95% CI 0.40–0.74; *p* = 0.0001). G-B followed by G maintenance demonstrated manageable toxicity, with a similar profile to B [12].

### Patient-reported HRQoL and disease-related symptoms

FACT-Lym questionnaire completion rates were determined based on the number of patients who completed the questionnaire divided by the number of evaluable patients. Patients were evaluable if they were alive at the point the visit was scheduled and were anticipated to complete study visit assessments, up until PD or follow-up completion. Completion rates of all scales were high in the G-B versus B arm during the induction phase (89.7 vs 88.6% at baseline; 77.7 vs 76.1% at EOI). During the maintenance/observation follow-up period, the completion rate in the B arm started to slightly reduce (G-B vs B 75.8 vs 62.8% at 6 months after EOI; 81.3 vs 67.2% at 12 months after EOI, and 76.1 vs 58.1% at 18 months after EOI).

### Absolute and change from baseline scores: ITT population

Mean and median baseline scores for each of the individual FACT-Lym questionnaire subscales, and of composite FACT-G, FACT-Lym TOI, and FACT-Lym TOT, were similar and well-balanced between arms. For each arm, mean scores were between 73 and 82% of the total range for each scale, with the exception of FWB which was the most impaired (64% of the total range).

**Fig. 1:**
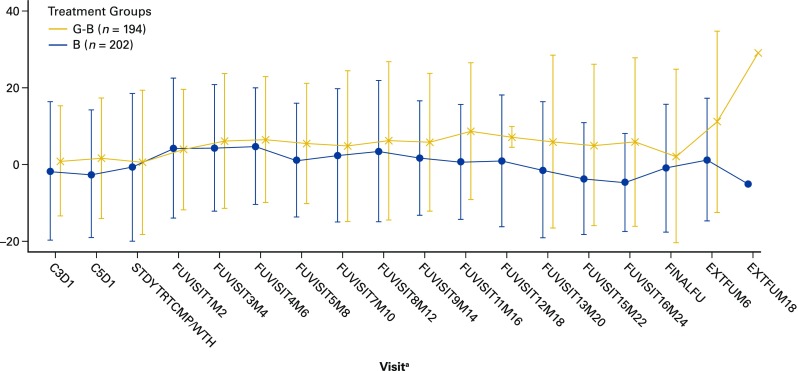
Mean change from baseline on FACT-Lym total score—ITT population. *B* bendamustine, *C* cycle, *D* day, *EXT* extended, *FACT-Lym* Functional Assessment of Cancer Treatment-Lymphoma questionnaire, *FU* follow-up, *G-B* obinutuzumab plus bendamustine, *M* month, *STDY* study, *TRTCMP* treatment completion, *WTH* early withdrawal. ^a^ For patients in the G-B arm, follow-up visits occurred during G maintenance treatment

Over the course of treatment, there were modest (primarily <5% of the baseline score) changes in both arms at each assessment in overall mean scores on all FACT-Lym questionnaire scales. The greatest improvements were observed on the FACT-Lym TOI and FACT-Lym TOT (Fig. 1) for both arms. In addition, average scores at each assessment were relatively similar between arms on the FACT-Lym LYMS, FACT-Lym TOI, and FACT-Lym TOT (Supplementary Table 1), with some tendency towards higher scores in the G-B arm post induction.

### Time to deterioration: ITT population

**Fig. 2:**
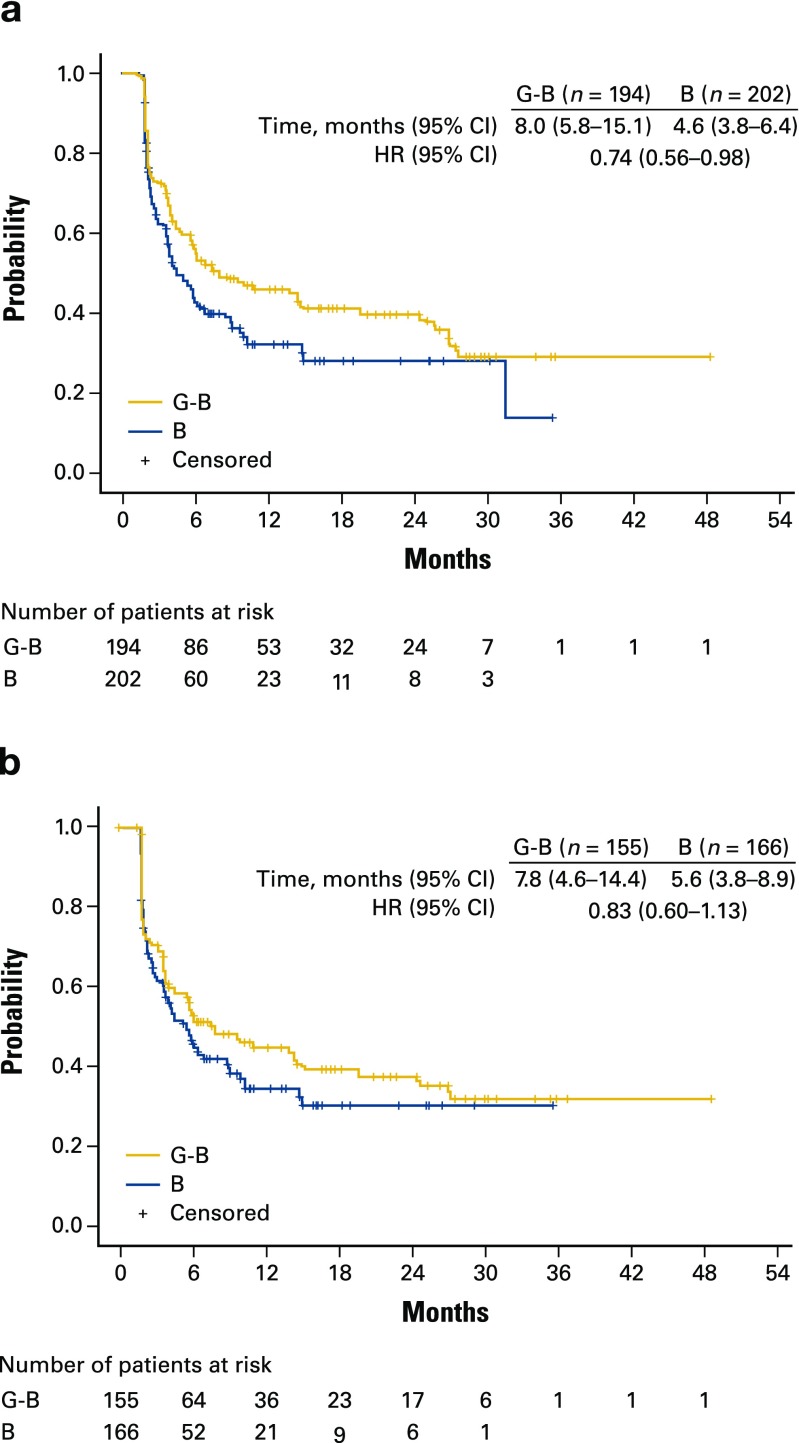
Kaplan-Meier plots of the FACT-Lym trial outcome index ≥6-point worsening from baseline in **a** the ITT population and **b** the FL subpopulation. *B* bendamustine, *CI* confidence interval, *FACT-Lym* Functional Assessment of Cancer Treatment Lymphoma questionnaire, *FL* follicular lymphoma, *HR* hazard ratio, *ITT* intent-to-treat

G-B treatment was associated with a delay in time to deterioration of FACT-Lym TOI scores, as defined by a ≥6-point worsening from baseline [18]. Median time to worsening was 8.0 months in the G-B arm and 4.6 months in B (Fig. 2a). The Kaplan-Meier estimated event-free rate for FACT-Lym TOI score deterioration (proportion of patients whose composite score had not worsened by ≥6 points from baseline) was higher in the G-B arm compared with B at 6 months (54.9 vs 44.0%, respectively) and 1 year (46.3 vs 32.1%, respectively), indicating a measure of clinical benefit for G-B.

The Kaplan-Meier plot of time to deterioration in FACT-Lym TOI showed that arms started to separate in favor of G-B at the time of the first scheduled post-baseline PRO assessment on D1 of C3 and remained separated until >18 months after trial entry when few patients remained at risk. Importantly, this includes the maintenance phase, where patients in the G-B arm received G and patients in the B arm were under observation only.

### Clinically meaningful improvement: ITT population

Similarly, a greater proportion of patients reported meaningful improvement on the FACT-Lym LYMS, FACT-Lym TOI, and FACT-Lym TOT in the G-B arm than B, at various time points throughout the study (Table 1). By selected treatment-assessment visits, the greatest difference between arms in FACT-Lym improvement rates was observed during the initial 2-year maintenance/follow-up period after EOI.

**Table 1:** Summary of meaningful improvement in FACT-Lym (ITT population and FL subpopulation)^a^

FACT-Lym questionnaire subscale (definition of meaningful improvement)^b^	ITT		FL	
	B (*n* = 202)	G-B (*n* = 194)	B (*n* = 166)	G-B (*n* = 155)
FACT-Lym LYMS (≥3-point increase)				
C5 and D1 (induction treatment) (%)	44/142 (31.0)	61/148 (41.2)	39/115 (33.9)	47/117 (40.2)
Follow-up visit 4 and 6 months post-EOI (%)	29/76 (38.2)	48/99 (48.5)	23/57 (40.4)	35/78 (44.9)
Follow-up visit 8 and 12 months post-EOI (%)	17/44 (38.6)	37/77 (48.1)	15/32 (46.9)	29/61 (47.5)
FACT-Lym TOI (≥6-point increase)				
C5 and D1 (induction treatment) (%)	32/143 (22.4)	52/149 (34.9)	28/115 (24.3)	40/118 (33.9)
Follow-up visit 4 and 6 months post-EOI (%)	22/77 (28.6)	43/99 (43.4)	17/58 (29.3)	32/78 (41.0)
Follow-up visit 8 and 12 months post-EOI (%)	12/44 (27.3)	37/77 (48.1)	10/32 (31.3)	28/61 (45.9)
FACT-Lym TOT (≥7-point increase)				
C5 and D1 (induction treatment) (%)	33/143 (23.1)	41/149 (27.5)	29/115 (25.2)	30/118 (25.4)
Follow-up visit 4 and 6 months post-EOI (%)	26/77 (33.8)	42/99 (42.4)	20/58 (34.5)	32/78 (41.0)
Follow-up visit 8 and 12 months post-EOI (%)	13/44 (29.5)	35/77 (45.5)	10/32 (31.3)	26/61 (42.6)

*B* bendamustine, *C* cycle, *D* day, *EOI* end of induction, *FACT-Lym* Functional Assessment of Cancer Treatment-Lymphoma questionnaire, *FACT-Lym LYMS* FACT-Lym lymphoma-specific subscale, *FACT-Lym TOI* FACT-Lym trial outcome index, *FACT-Lym TOT* FACT-Lym total score, *FL* follicular lymphoma, *G-B* obinutuzumab plus bendamustine, *ITT* intent-to-treat

^a^Values are *n*/total *n* (%); scale score increases of ≥3, ≥6, and ≥7 points are reflective of the amounts of change that represent a clinically meaningful improvement in patient HRQoL

^b^For patients in the G-B arm, follow-up assessments occurred during G maintenance treatment

### FL subpopulation

Most patients (321/396; 81%) in the ITT population had a FL diagnosis, and it was anticipated that outcomes in the FL subpopulation would be consistent with the ITT population. However, since the ITT population included non-FL patients with different prognoses than FL patients, the analyses were repeated for the FL subpopulation only. As with the ITT population, there were no notable differences between treatment arms in any of the average scores on the FACT-Lym questionnaire subscales at baseline, over time during the treatment period, and at follow-up.

G-B treatment suggested a longer delay in the time to deterioration of FACT-Lym TOI score. Median time to worsening of FACT-Lym TOI was 7.8 months in the G-B arm and 5.6 months in B (Fig. 2b). Kaplan-Meier estimated event-free probabilities for FACT-Lym TOI score deterioration were higher in the G-B arm compared with B at 6 months (52.8 vs 46.7%, respectively) and 1 year (45.0 vs 34.7%, respectively), indicating a degree of clinical benefit for the G-B arm. A higher proportion of patients in the G-B arm also had improvement in their FACT-Lym questionnaire scores during treatment and follow-up (Table 1).

## Discussion

For R-refr iNHL patients, G-B treatment provides an important option by achieving an increased PFS over standard of care with B alone [12]. Of equal importance is understanding the associated impact on HRQoL.

In GADOLIN, individuals in both treatment arms reported slightly impaired HRQoL prior to treatment, with the greatest impairment being observed on the FWB scale whose items include those focused on ability to work, sleep, and enjoy life and fun activities. Scores on the PWB, FWB, SWB, and EWB scales at baseline were roughly 2–3 points lower than the general population averages [20], 1–3 points lower than scores reported by a disease-free NHL (all subtypes) sample [21], but 0–7 points higher than a group of active disease-relapsed FL patients [13]. This observation suggests that patients experienced a clinically meaningful lower HRQoL as compared to the general population, but higher HRQoL than published data on patients with relapsed disease. As 15/34 (44%) of the active disease-relapsed patients in the Pettengell study were receiving chemotherapy at the time of assessment, it is possible that the difference observed is the result of the unspecified chemotherapy regimen received by patients adversely impacting their HRQoL [13].

During the study, there was minimal change in the overall group average scale scores in both arms. These minimal changes from baseline are consistent with the lack of a significant decline in FACT-Lym TOT, FACT-Lym questionnaire, or the European Organization for Research and Treatment of Cancer QLQ-C30 subscale scores during the study period reported in frontline and previously treated rituximab-exposed iNHL patients [22, 23]. While no clear improvement in HRQoL was observed with G-B treatment, when looking at the overall sample, results showed no evidence to suggest that G-B treatment reduced patient-reported HRQoL—an important factor in refractory iNHL patients in need of alternative treatment.

Although there were no differences between arms in the overall sample average scores, there were differences in patients who reported clinically meaningful changes over the course of the study: a longer time to clinically meaningful deterioration in lymphoma-related HRQoL in the G-B arm compared with B alone. In addition, G-B followed by G maintenance resulted in a greater proportion of patients reporting a meaningful improvement in HRQoL throughout the study. This improvement occurred in the G-B arm even during induction, despite similar clinical response rates [12], and the benefit was sustained over time despite the delivery of additional G treatment.

In the primary GADOLIN manuscript, subgroup analyses of efficacy demonstrated that the benefit of G-B was seen in the majority of subgroups tested, including FL (81% of ITT), in whom the stratified HR for IRC-assessed PFS in the G-B arm relative to B alone was 0.48 (95% CI 0.34–0.68) [12]. It was important to determine whether the clinical benefit observed in FL patients was consistent for HRQoL, which would provide additional support for the benefit of G-B treatment. As noted, results of HRQoL analysis were similar between the overall ITT population and FL subpopulation.

The primary limitation of this study is the decreased number of patients completing the HRQoL questionnaires over time. Although completion rates in the B arm started to reduce, the degree of missing data among those who were progression free and evaluable to complete the questionnaires was not markedly different between treatment arms. Therefore, it is likely that the scores are a reflection of the HRQoL of treated patients. At 18 months from EOI, 75 evaluable patients remained in the G-B arm and 25 in B. This was due to a combination of PD, death, study discontinuation, and the study being reported after reaching statistically significant results in PFS at interim analysis. If completion rates are examined based on the proportion of FACT-Lym questionnaire data missing, due to attrition and non-compliance, the difference between arms is more pronounced than due to compliance alone. Specifically, the proportion of data missing was higher in the B arm during follow-up/maintenance (61% in B arm at 6 months after EOI; 76% at 12 months after EOI; 81% at 18 months after EOI) than the corresponding numbers for G-B (41, 52, 57%, respectively), which is in line with the higher attrition rates due to PD and death in the B arm. A further limitation is that after PD, HRQoL questionnaires were only collected at study treatment/follow-up termination visit, and <50% of patients with PD completed them, which could potentially have biased the results. The proportion of patients that were event free with respect to progression or death in the G-B versus B arm was 76.6 versus 57.4% after 1 year and 59.4 versus 35.4% after 1.5 years from randomization. If we consider progression to have an adverse impact on HRQoL data, then data reported only from the non-progressed patients are likely an overestimate of the true HRQoL. As the progression rate was significantly greater in the B arm (and thus the relapse-free follow-up shorter), this could be interpreted as the change from baseline in HRQoL is biased upwards to a greater extent in the B arm compared with G-B.

As the aim of treatment for R-refr iNHL patients is to maximize PFS, maintain HRQoL, and minimize treatment-related morbidity, the results of GADOLIN highlight the benefit that G-B treatment confers over B alone in some patients, while maintaining pre-treatment levels of HRQoL. There was no evidence to suggest that G-B treatment reduced patient-reported HRQoL, and in a proportion of patients, resulted in meaningful improvements in lymphoma-related symptoms. Our results suggest that improved PFS is not at the expense of an increase in treatment-related toxicity that could lead to a reduction in a patient’s HRQoL.

## Electronic supplementary material

ESM 1 (PDF 191 kb)
