# Supplementary material for: Health-related quality of life and symptoms in patients with rituximab-refractory indolent non-Hodgkin lymphoma treated in the phase III GADOLIN study with obinutuzumab plus bendamustine versus bendamustine alone
Source: Ann Hematol. 2016 Nov 30;96(2):253–9. doi: 10.1007/s00277-016-2878-5 (PMC5226995; doi:10.1007/s00277-016-2878-5)
Supplement: Supplementary file 1 — (PDF 191 kb) [file 277_2016_2878_MOESM1_ESM.pdf]

Supplementary Material

Health-related quality of life and symptoms in patients with rituximab-refractory, indolent non-Hodgkin lymphoma treated in the phase III GADOLIN study with obinutuzumab plus bendamustine versus bendamustine alone

Annals of Hematology

Bruce D. Cheson[1], Peter C. Trask[2], John G. Gribben[3], Natalie Dimier[4], Eva Kimby[5], Pieternella J. Lugtenburg[6], Catherine Thieblemont[7], Elisabeth Wassner-Fritsch[8], Aino Launonen,[8] and Laurie H. Sehn[9]

Corresponding author: Bruce D. Cheson

Georgetown University Hospital, Lombardi Comprehensive Cancer Center, 3800 Reservoir Road NW, Washington, DC 20007

E-mail: bdc4@georgetown.edu

Supplementary Table 1 FACT-Lym scores by visit: ITT population

FACT-Lym questionnaire subscale[a–c]	B (n = 202)		G-B (n = 194)	
	Mean (SD)	Mean change from baseline (SD)	Mean (SD)	Mean change from baseline (SD)
PWB				
Baseline	22.63 (5.16)	N/A	22.72 (4.65)	N/A
C5, D1	20.96 (5.28)	-1.99 (5.07)	21.97 (5.20)	-0.77 (4.22)
Study treatment completion/early withdrawal	22.26 (5.11)	-0.96 (5.23)	22.16 (5.58)	-0.63 (4.71)
Follow-up visit 4, 6 months post-EOI	24.16 (3.40)	0.31 (3.74)	24.34 (3.64)	0.77 (3.62)
Follow-up visit 8, 12 months post-EOI	24.36 (3.86)	0.50 (4.26)	24.60 (4.20)	0.66 (4.60)
FWB				
Baseline	18.00 (6.18)	N/A	17.84 (6.07)	N/A
C5, D1	17.62 (5.80)	-0.88 (5.04)	18.58 (6.12)	0.67 (5.48)
Study treatment completion/early withdrawal	17.79 (5.96)	-0.58 (5.68)	18.09 (6.44)	0.00 (5.35)
Follow-up visit 4, 6 months post-EOI	19.41 (5.10)	0.81 (4.65)	19.95 (5.73)	1.58 (5.05)
Follow-up visit 8, 12 months post-EOI	19.34 (5.19)	1.11 (4.82)	19.95 (5.96)	1.96 (6.63)
SWB				

Baseline	22.14 (5.58)	N/A	22.17 (5.48)	N/A
C5, D1	21.78 (5.35)	-0.82 (3.45)	21.85 (4.69)	-0.39 (4.40)
Study treatment completion/early withdrawal	21.53 (5.48)	-1.06 (4.69)	21.56 (5.86)	-0.91 (3.62)
Follow-up visit 4, 6 months post-EOI	22.30 (5.47)	-0.14 (2.84)	22.11 (5.30)	-0.07 (4.81)
Follow-up visit 8, 12 months post-EOI	23.11 (3.75)	0.13 (2.31)	21.95 (5.47)	-0.14 (5.53)
EWB				
Baseline	17.43 (4.44)	N/A	17.73 (4.33)	N/A
C5, D1	18.18 (4.19)	0.32 (3.11)	18.28 (4.72)	0.50 (3.59)
Study treatment completion/early withdrawal	18.26 (4.05)	0.53 (3.59)	18.24 (4.65)	0.48 (3.97)
Follow-up visit 4, 6 months post-EOI	18.74 (3.38)	0.94 (3.42)	19.18 (3.96)	0.97 (3.29)
Follow-up visit 8, 12 months post-EOI	18.07 (3.95)	-0.08 (3.98)	19.14 (4.14)	0.84 (3.01)
FACT-Lym LYMS				
Baseline	44.88 (9.56)	N/A	45.55 (9.18)	N/A
C5, D1	46.15 (8.82)	0.88 (6.87)	46.73 (9.84)	1.26 (6.10)
Study treatment completion/early withdrawal	47.36 (9.33)	1.88 (7.18)	46.13 (10.24)	0.79 (7.99)
Follow-up visit 4, 6 months post-EOI	48.32 (8.48)	2.57 (6.65)	50.32 (7.74)	3.45 (6.36)
Follow-up visit 8, 12 months post-EOI	48.36 (7.80)	2.53 (7.35)	50.41 (7.79)	3.23 (6.50)

FACT-G				
Baseline	80.07 (15.87)	N/A	80.61 (16.19)	N/A
C5, D1	78.41 (15.88)	-3.28 (11.72)	80.56 (16.92)	-0.04 (11.37)
Study treatment completion/early withdrawal	79.45 (15.46)	-2.23 (13.80)	79.91 (18.33)	-1.08 (11.87)
Follow-up visit 4, 6 months post-EOI	84.91 (13.38)	2.28 (10.10)	85.62 (15.21)	3.33 (11.52)
Follow-up visit 8, 12 months post-EOI	84.74 (12.84)	1.63 (11.44)	85.53 (15.86)	2.98 (15.28)
FACT-Lym TOI				
Baseline	84.79 (19.01)	N/A	84.53 (19.03)	N/A
C5, D1	84.40 (17.76)	-1.55 (15.60)	86.82 (19.82)	2.23 (14.34)
Study treatment completion/early withdrawal	86.32 (18.95)	-0.40 (17.90)	85.82 (20.75)	0.44 (16.73)
Follow-up visit 4, 6 months post-EOI	90.44 (17.52)	3.34 (12.36)	94.60 (15.79)	6.56 (13.08)
Follow-up visit 8, 12 months post-EOI	92.07 (14.95)	4.14 (14.46)	94.96 (16.10)	6.92 (16.37)
FACT-Lym TOT				
Baseline	124.86 (23.63)	N/A	126.00 (23.96)	N/A
C5, D1	124.48 (23.12)	-2.37 (16.63)	127.26 (25.93)	1.47 (15.44)
Study treatment completion/early withdrawal	126.99 (23.40)	-0.11 (18.91)	125.96 (27.31)	0.25 (18.50)
Follow-up visit 4, 6 months post-EOI	133.36 (20.38)	5.08 (14.88)	135.89 (21.92)	6.85 (15.59)

Follow-up visit 8, 12 months post-EOI	133.11 (19.61)	4.19 (17.76)	135.97 (22.53)	6.41 (20.02)

B bendamustine, *C* cycle, *D* day, *EOI* end of induction, *EWB* emotional well-being, *FACT-Lym* Functional Assessment of Cancer Treatment-Lymphoma questionnaire, *FACT-G* FACT-General, *FACT-Lym LYMS* FACT-Lym lymphoma-specific subscale, *FACT-Lym TOI* FACT-Lym Trial Outcome Index, *FACT-Lym TOT* FACT-Lym total score, *FWB* functional well-being, *G-B* obinutuzumab plus bendamustine, *ITT* intent-to-treat, *PD* progression of disease, *PRO* patient-reported outcome, *PWB* physical well-being, *SWB* social/family well-being

[a] Patients followed the same schedule of PRO assessments during induction treatment and follow-up regardless of treatment arm, and no PRO assessment was scheduled after PD

[b] The FACT-Lym questionnaire was administered on D1 of C1, 3 and 5 during treatment, at the EOI treatment and bi-monthly for 2 years (where non-progressing G-B patients received G maintenance and B patients were observed), and annually during extended follow-up until PD

[c] For patients in the G-B arm, follow-up assessments occurred during G maintenance treatment
